# Rates of adherence, adherence measurement, and support services for children and adolescents living with HIV followed in global sites of the International Epidemiology Databases to Evaluate AIDS (IeDEA)

**DOI:** 10.1186/s12887-025-05939-4

**Published:** 2025-10-02

**Authors:** Rachel C. Vreeman, Constantin T. Yiannoutsos, Andrew Edmonds, Valériane Leroy, Geoffrey Fatti, Pope Kosalaraksa, Jorge Pinto, Beverly Musick, Winstone Nyandiko, Christelle Twizere, Madeleine Amorissani-Folquet, Safari Mbewe, Fernando Mejia, Michael L. Scanlon, Roxanne Martin, Kara Wools-Kaloustian

**Affiliations:** 1 Department of Global Health and Health Systems Design, Arnhold Institute for Global Health, Icahn School of Medicine at Mount Sinai, 1216 Fifth Avenue, 5 Floor, New York, NY 10029 USA; 2 Department of Epidemiology and Biostatistics, City University of New York, School of Public Health and Health Policy, New York, USA; 3 Department of Epidemiology, Gillings School of Global Public Health, University of North Carolina at Chapel Hill, Chapel Hill, NC USA; 4 Inserm, University of Toulouse, Toulouse, France; 5 Division of Epidemiology and Biostatistics, Department of Global Health, Faculty of Medicine and Health Sciences, Stellenbosch University, Cape Town, South Africa; 6 Division of Infectious Diseases, Department of Pediatrics, Faculty of Medicine, Khon Kaen University, Khon Kaen, Thailand; 7 Division of Immunology School of Medicine, Federal University of Minas Gerais, Belo Horizonte, MG Brazil; 8 Department of Biostatistics and Health Data Science, Indiana University School of Medicine, Indiana University, Indianapolis, IN USA; 9 Moi University College of Health Sciences, Eldoret, Kenya; 10 Academic Model Providing Access to Healthcare (AMPATH), Eldoret, Kenya; 11 Centre National de Référence en Matière de VIH/SIDA (CNR), Bujumbura, Burundi; 12 Pediatric Department, Centre Hospitalier Universitaire de Cocody, Abidjan, Côte d’Ivoire; 13 Lighthouse Trust, Lilongwe, Malawi; 14 Universidad Peruana Cayetano Heredia, Instituto de Medicina Tropical Alexander Von Humboldt, Lima, Peru; 15 Indiana University Center for Global Health Equity, Indiana University, Indianapolis, IN USA; 16 Department of Medicine, Indiana University School of Medicine, Indianapolis, IN USA

**Keywords:** Adherence, Adolescents, Children, Health systems, HIV care continuum

## Abstract

**Introduction:**

Supporting and improving antiretroviral therapy (ART) adherence and preventing the evolution of HIV drug resistance remain major challenges for children and adolescents living with HIV globally. In a large global HIV clinical data consortium, we sought to describe how global HIV care programs measure and support pediatric ART adherence, as well as patient-level measures of adherence.

**Methods:**

We prospectively collected site-level data between June 2014-March 2015 using a site assessment survey and retrospectively examined patient-level data collected during routine clinical care, to provide a comprehensive assessment of pediatric ART adherence across the International Epidemiology Databases to Evaluate AIDS (IeDEA) cohort, in six global regions. All regions capturing patient-level data on adherence by any measure for children living with HIV aged less than 14 years between 2000–2015 were asked to contribute data. ART adherence was conceptualized as a binomial variable of “good” (greater than 90%) or “poor” (90% or less) adherence per visit.

**Results:**

Clinical staff from 180 pediatric sites in 45 countries completed the adherence survey. Clinician adherence assessment (used at 87% of sites) and pharmacy refills (86% of sites) were the most common adherence measurement methods used globally. Counseling focused on adherence was the most commonly available support service, (94% of sites). 28,664 pediatric patients had at least one adherence measurement, to be included for adherence analyses. In East Africa and Southern Africa, higher baseline CD4 counts were associated with a greater likelihood of viral suppression; however, in Central and South America and the Caribbean (CCASAnet) and Asia–Pacific, we did not find a consistent relationship between baseline CD4 count and the likelihood of viral suppression. We found evidence that very young children (< 2 years of age), older children (> 10 years of age), and males were less likely to experience viral suppression in East Africa and Southern Africa.

**Conclusion:**

These findings indicate that the majority of global pediatric HIV care services routinely measure ART adherence for children living with HIV; however, few sites use objective or validated measures. Identifying subgroups of children and youth at highest risk for non-adherence allows care programs to target those most in need of adherence support or resistance monitoring.

**Supplementary Information:**

The online version contains supplementary material available at 10.1186/s12887-025-05939-4.

## Introduction

Access to antiretroviral therapy (ART) gives the world’s 1.7 million children living with HIV a chance to grow into adolescence and adulthood [1]. Sustaining high levels of adherence to ART remains a central challenge for long-term HIV care, both to avoid HIV-related complications and to prevent onward HIV transmission [2–4].

Though 90% of the world’s children and adolescents living with HIV live in resource-limited settings [1], we still do not know the most effective strategies to improve long-term ART adherence for children and adolescents in resource-limited settings. There is limited evidence on how adherence varies in pediatric populations over time or on the best clinical practices to sustain adherence [5, 6]. Moreover, reliable, valid measurement of pediatric adherence and addressing non-adherence remain challenges for healthcare systems [5, 7].

We sought to describe how global HIV care programs measure and support pediatric ART adherence as programs entered the “Treat All” era of initiating ART for all children under 15 years and living with HIV. We collected prospective survey data from clinical HIV care sites within the International Epidemiology Databases to Evaluate AIDS (IeDEA) consortium (www.iedea.org.) We also aimed to describe individual patient data on pediatric ART adherence among children living with HIV and receiving care at these sites. By investigating both the site-level and the individual-level data, we sought to describe and elucidate the global context of ART adherence for children with HIV and their families, even in the face of changing regimens and treatment strategies. In addition, we sought to assess the impact of pediatric non-adherence on clinical outcomes of treatment failure, losses to follow-up and mortality.

## Methods

This global pediatric adherence assessment employed analyses at both the site-level and patient-level to provide a comprehensive assessment of pediatric ART adherence across the large global cohort followed in IeDEA. We conducted this evaluation within six global regions of the IeDEA consortium caring for children living with HIV (Asia–Pacific; Central and South America and the Caribbean (CCASAnet); West Africa, Central Africa, East Africa, and Southern Africa). Funded by multiple institutes of the U.S. National Institutes of Health since 2006, the IeDEA consortium unites HIV clinical care programs caring for over 2.2 million people living with and at risk for HIV, within 44 countries, to compile observational, globally diverse clinical cohort data.

To gather site-level data, a specific web-based survey (REDCap) was used in which individual HIV care sites from across the six global regions completed survey questions about their methods of adherence measurement for children and adolescents, support services for adherence, training related to pediatric adherence and HIV disclosure, and relevant pediatric care system characteristics [8]. Surveys were completed between June 2014-March 2015. The survey also included case-based assessments of patient management to further clarify sites’ available treatment options and strategies for diagnosing treatment failure, identifying and addressing children’s adherence problems, and employing interventions.

To examine individual-level factors associated with medication adherence and the connection between non-adherence and treatment failure, losses to follow-up and mortality, a retrospective analysis of existing pediatric patient-level data was also conducted. Children were eligible for inclusion if they were living with HIV, on ART, less than 14 years of age at enrollment, followed at a participating IeDEA site, and had at least 1 adherence measure after initiation of ART. All regions capturing patient-level data for children meeting these eligibility criteria were asked to contribute data from 2000 to 2015.

ART adherence was conceptualized as a binomial variable of “good” (greater than 90%) or “poor” (90% or less) adherence per visit, with variable creation dependent on the adherence measure. For recall measures, “good adherence” for a visit was defined as reporting that “all” doses were taken or that “no” doses are missed, or a similar dichotomization based on the response options to indicate > 90% of doses taken. Selection of 90% adherence was consistent with the standard used in prior studies of similar populations; previous studies have also shown that below 90% of adherence, the risks for HIV virological rebound and drug resistance are increased [7, 9, 10]. For adherence measured through pill counts, if pill counts were recorded categorically, the categorical determinations were categorized as “good adherence” or “poor adherence.” For quantitative pill counts, pill count or liquid volume measures were converted into estimates of whether > 10% of doses were missed. Similar strategies were used to dichotomize both qualitative and quantitative records of pharmacy refills.

We describe rates of ART adherence, virologic suppression (defined as HIV RNA < 400 copies/mL), and demographic and clinical characteristics of the participating IeDEA sites’ pediatric populations. We used a smoothed line (LOESS) and associated 95% confidence interval to visualize trends of good adherence over time. The smoothing line was applied to the raw proportions of good adherence over time binned in 8-week intervals. The fit was weighted for the total number of adherence measures available in each binned time point, so that temporally extreme time points with few observations would not dominate the curve fit. Default smoothing coefficients were used as prescribed by the SAS software (SAS Institute, Cary NC), i.e., a smoothing coefficient $\lambda =0.6$, was used.

The association of both clinic-level and individual-level variables with ART adherence were considered. We performed analyses to evaluate factors predicting ART adherence and adherence-related clinical outcomes: treatment failure, mortality, and loss to follow-up (defined as having the last visit greater than 6 months prior to death or database closure, without documented transfer to another clinic). We performed multivariable logistic regression to assess the independent association between odds of ART non-adherence and patient-level variables (age, sex, and CD4 count). As not all regions obtained measures of good adherence, we analyzed viral suppression (dichotomized as < 400 copies/mL) as a proxy measure of good adherence. We performed two separate Cox proportional hazards regression models, one with loss to follow-up and one with mortality as the outcome, to assess the independent effect of time-updated good adherence (again using the dichotomous measure of viral suppression as a proxy), along with sex plus CD4 count and age at ART initiation. All analyses were stratified by region because of differences in data availability and types of programs in each region.. A *p*-value of < 0.05 was considered statistically significant. All analyses were performed with SAS version 9.4 (SAS Institute, Cary NC).

## Results

### Site-level characteristics

**Fig. 1:**
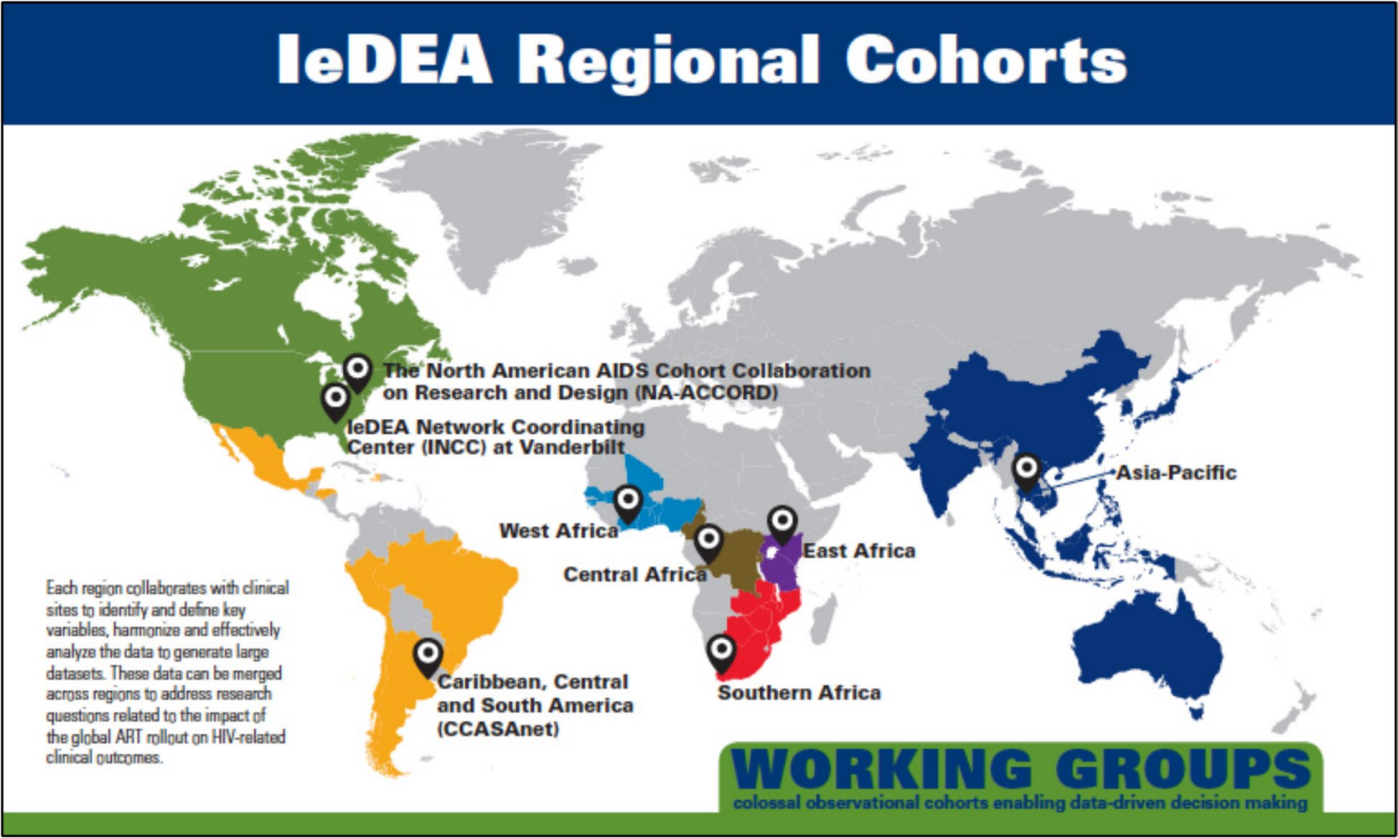
Map of IeDEA Regional Cohorts

Clinical staff from 180 pediatric sites in 45 countries completed the adherence survey (Fig. 1). The sites were in the Asia–Pacific (*n* = 16 sites; 4,357 children), the Caribbean, Central and South America [CCASAnet] (*n* = 7; 1,746 children), Central Africa (*n* = 18; 906 children), East Africa (*n* = 33; 12,218 children), Southern Africa (*n* = 95; 45,641 children), and West Africa (*n* = 11; 8,932 children). Most sites managed both adults and children (82%).

### Site-level adherence measurement strategies

Clinician adherence assessment (used at 87% of sites) and pharmacy refills (86% of sites) were the most common adherence measurement methods used globally (Table 1). All sites used at least one measure. Other common methods were structured recall items (one recall item, any report time period) (77%) and targeted HIV viral loads used when clinicians were concerned about adherence (75%). Instruments with multiple adherence question items were used in 61% of sites, and few sites used validated measures. Pill counts were used in 40% of sites. Twelve global sites (7%) used drug levels, and 4 sites (2%) used electronic dose monitoring, but these measurement strategies were also noted to be accessible primarily through research studies. The percentage of sites in a region using routine viral load monitoring to assess adherence ranged from 13–79%.

**Table 1:** ART adherence measurement methods used for children by Global IeDEA sites

Adherence Measure	Asia–Pacific (*N* = 16)	CCASAnet (*N* = 7)	Central Africa (*N* = 18)	East Africa (*N* = 33)	Southern Africa (*N* = 95)	West Africa (*N* = 11)	Total (*N* = 180 Sites)
Clinician assessment	13 (81%)	7 (100%)	13 (72%)	22 (67%)	93 (98%)	9 (82%)	157 (87%)
Structured recall (one recall item, any time period)	7 (44%)	3 (43%)	14 (78%)	20 (61%)	87 (92%)	8 (73%)	139 (77%)
Instrument with multiple adherence questions	4 (25%)	0 (0%)	7 (39%)	16 (48%)	78 (82%)	5 (45%)	110 (61%)
Pill counts	10 (63%)	2 (29%)	14 (78%)	27 (82%)	13 (14%)	6 (55%)	72 (40%)
Liquid measures^a^	5 (31%)	2 (29%)	12 (67%)	8 (24%)	8 (8%)	5 (45%)	40 (22%)
Pharmacy refill	7 (44%)	4 (57%)	16 (89%)	27 (82%)	90 (95%)	10 (91%)	154 (86%)
Electronic dose monitoring^b^	0 (0%)	0 (0%)	0 (0%)	1 (3%)	3 (3%)	0 (0%)	4 (2%)
Plasma Drug Levels^b^	0	0	1 (6%)	10 (30%)	1 (1%)	0 (0%)	12 (6%)
Viral load monitoring at routine intervals	10 (63%)	5 (71%)	13 (72%)	26 (79%)	12 (13%)	8 (73%)	74 (41%)
Viral load if suspect non-adherence	9 (56%)	3 (43%)	14 (78%)	16 (48%)	86 (91%)	7 (64%)	135 (75%)

^a^Liquid measures considered an equivalent to a pill count, but an estimation of liquid formulation of medication remaining (specific to pediatric liquid formulations.)

^b^Both electronic dose monitoring and plasma drug levels within these settings noted to be available primarily through research studies

Available adherence support services varied widely across sites (Table 2). Counseling focused on adherence was the most commonly available support service, reported as available at 94% of sites or at 89% to 100% of sites per region. Nutritional support and case management were also frequently reported as support services, available at 88% of sites. Home visits were widely employed in East Africa and Southern Africa sites (88% to 94%), but less frequently in the Asia–Pacific and CCASAnet sites (57% to 63%). Adherence support requiring additional technology was not generally available globally; however, pill boxes were available at 19% of sites and SMS adherence reminders were available at 11% of sites.

**Table 2:** ART adherence support available at global IeDEA sites

Adherence Measure	Asia–Pacific (*N* = 16)	CCASAnet (*N* = 7)	Central Africa (*N* = 18)	East Africa (*N* = 33)	Southern Africa (*N* = 95)	West Africa (*N* = 11)	Total (*N* = 180 Sites)
Pill boxes	12 (75%)	2 (29%)	1 (6%)	9 (27%)	8 (8%)	2 (18%)	34 (19%)
Counseling (any)	16 (100%)	7 (100%)	16 (89%)	32 (97%)	89 (94%)	10 (91%)	170 (94%)
Case management	13 (81%)	5 (71%)	13 (72%)	28 (85%)	89 (94%)	10 (91%)	158 (88%)
Educational classes	9 (56%)	3 (43%)	15 (83%)	29 (88%)	89 (94%)	8 (73%)	153 (85%)
Support groups							
For caregivers	13 (81%)	6 (86%)	12 (67%)	28 (85%)	88 (93%)	8 (73%)	155 (86%)
For children	9 (56%)	5 (71%)	13 (72%)	26 (79%)	92 (97%)	9 (82%)	154 (86%)
Peer adherence supporters	3 (19%)	2 (29%)	10 (56%)	25 (76%)	87 (92%)	5 (45%)	132 (73%)
Home visits	10 (63%)	4 (57%)	17 (94%)	25 (76%)	81 (85%)	8 (73%)	145 (81%)
SMS adherence reminders	4 (25%)	2 (29%)	1 (6%)	9 (27%)	2 (2%)	1 (9%)	19 (11%)
Nutrition support	12 (75%)	4 (57%)	14 (78%)	32 (97%)	88 (93%)	9 (82%)	159 (88%)

### Site-level case management and ART availability

Overall, 155 of 180 sites (86%) reported having available second-line regimens to address treatment failure in a 4-month-old, while 94% and 98% of sites reported available second-line regimens to address treatment failure in a 3-year-old and 7-year-old, respectively. Sites in urban areas and larger hospitals (regional, provincial, or University) were more likely to report having available second-line regimens; sites in the Central Africa, CCASAnet, or East Africa regions were less likely. Significantly fewer sites (only 10% overall) reported having access to third-line regimens, particularly sites in the Central, East, South, and West Africa regions. To guide clinical decision-making, most sites reported access to targeted viral load testing (95%) and CD4 monitoring (95%), with fewer sites (68%) reporting access to viral resistance testing.

### Patient-level characteristics and outcomes

Patient-level data were available for 50,532 pediatric patients in the Asia–Pacific (*n* = 16 sites; 4,357 children), CCASAnet (*n* = 7; 1,742 children), Central Africa (*n* = 18; 906 children), East Africa (*n* = 33; 11,898 children) and Southern Africa (*n* = 95; 31,629 children) (Table 3). Patient-level data for this period from West Africa were not available. The global median age at enrollment was 4.3 years (IQR 1.5,8.0) and at ART start was 4.9 years (IQR 1.8,8.6). The sites in CCASAnet and Southern Africa both enrolled and started children on ART at younger ages than other regions. Median CD4 percentage and weight for age z-score at ART start was 15.7 (IQR 10.0,23.0) and −1.9 (IQR −3.0,−0.9), respectively. Children at sites in CCASAnet tended to have higher CD4 percentage and weight for age and height for age z-scores at enrollment and ART start compared to children in other regions. The majority of pediatric patients (81%) were on an NNRTI-based first line regimen, with only 13% having a PI-based first line regimen. Children at sites in CCASAnet (24%) and Southern Africa (18%) regions were more likely to have a PI-based first-line regimen. Among the pediatric patients meeting the inclusion criteria between 2000–2015, at assessment in 2015, 6.5% had died and 12.8% were lost to follow up, with the highest proportion of mortality and lost to follow up from the CCASAnet region. Higher CD4 count was associated with significantly lower likelihood of mortality across all regions. Younger age (children aged between 2–10 years compared to ≥ 10 years) was associated with approximately half the mortality (15%−50% reduction in mortality hazard) in all cases.

**Table 3:** Pediatric patients with individual-level data

Characteristic	Total *N* = 50,532	Asia–Pacific *N* = 4,357	CCASAnet *N* = 1,742	Central Africa *N* = 90^f^	East Africa *N* = 11,898	Southern Africa *N* = 31,629
N(%)						
Female	25,272 (50)	2,096 (48)	911 (52)	455 (50)	6,073 (51)	15,737 (50)
Deceased	3,295 (6.5)	342 (8)	259 (15)	35 (4)	686 (6)	1,973 (6)
Lost to Follow-up^a^	6,461 (12.8)	431 (10)	596 (34)	229 (25)	2,533 (21)	2,672 (8)
First Line Regimen						
NNRTI-based	41,075(81.3)	3,712 (85)	1,084 (62)	843 (93)	10,978(92)	24,458(77)
PI-based	6,434 (12.7)	245 (6)	423 (24)	58 (6)	170 (1)	5,538 (17)
Triple NRTI	419 (0.8)	96 (2)	71 (4)	3 (0)	117 (1)	132 (0)
INSTI-based	5 (0)	3 (0)	0 (0)	0 (0)	0 (0)	2 (0)
Unknown	2,599 (5.2)	301 (7)	164 (9)	2 (0)	633 (5)	1,499 (5)
Median (IQR)						
Age at enrollment	4.28 (1.54,8.03)	4.6 (1.9,7.7)	3.2 (1.0,7.1)	4.5 (1.7,8.3)	4.8 (2.2,8.2)	4.0 (1.4,8.0)
Age at ART start	4.88 (1.83,8.61)	5.0 (2.2,8.1)	4.1 (1.4,8.3)	4.9 (2.0,8.9)	5.9 (2.8,9.3)	4.4 (1.6,8.4)
CD4 count at ART start (cells/µl)^b^	266 (108,467)^c^	130 (28,308)	281 (104,499)	297 (116,528)	301 137,549)	268 (125,451)
CD4% at ART start^d^	15.7 (10.0,23.0)^c^	15 (7,22)	18 (13,27)	15 (10,21)	15 (11,22)	16 (10,23)
Weight for Age Z-score at enrollment	−1.9 (−3.1,−0.9)^e^	−2.2 (−3.3,−1.2)	−1.5 (−2.8,−0.6)	−2.1 (−3.1,1.1)	−1.8 (−2.9.−0.8)	−2 (−3.2,−0.9)
Weight for Age Z-score at ART start	−1.9 (−3.0,−0.9)^e^	−2.2 (−3.4,−1.2)	−1.6 (−2.8,−0.6)	−1.9 (−2.9,0.9)	−1.7 (−2.8,−0.7)	−1.9 (−3.2,−0.9)
Height for Age Z-score at enrollment	−2.4 (−3.5,−1.3)^f^	−2.4 (−3.3,−1.4)	−1.7 (−2.7,−0.8)	−2.2 (−3.2,1.2)	−2.2 (−3.3,−1.1)	−2.5 (−3.6,−1.4)
Height for Age Z-score at ART start	−2.4 (−3.5,−1.3)^f^	−2.4 (−3.3,−1.4)	−1.7 (−2.7,−0.8)	−2 (−3,−1.1)	−2.2 (−3.3,−1.1)	−2.5 (−3.6,−1.5)

^a^Lost to follow-up defined as > 6 months without a visit and not known to have transferred to another clinic

^b^Patients >= 5 years old (*N* = 15,470)

^c^Closest within 182 days prior and 14 days post ART initiation

^d^Patients < 5 years old (*N* = 10,955)

^e^Closest within 90 days prior and 14 days post ART initiation

^f^Closest within 90 days prior/post

### Patient-level adherence measures and outcomes

A total of 28,664 pediatric patients had the minimum required data—i.e. at least one adherence measurement—to be included for adherence analyses. Among Asia–Pacific sites, 3,114 pediatric patients (71%) were included using viral load adherence measurement with a median of 6 adherence measures per patient and 175 days between adherence measures. Among CCASAnet sites, 957 pediatric patients (55%) were included using viral load adherence measurement with a median of 15 adherence measures per patient and 126 days between measures. Among Central Africa sites, 905 pediatric patients (99%) were included using qualitative assessment of adherence with a median of 21 adherence measures per patient and 33 days between measures. Among East Africa sites, 11,898 pediatric patients (100%) were included using multiple adherence measures (7 different measures) with a median of 25 adherence measures per patient and 35 days between measures. Finally, among Southern Africa sites, 11,962 pediatric patients (38%) were included using viral load measurement with a median of 4 adherence measures and 173 days between measures.

**Fig. 2:**
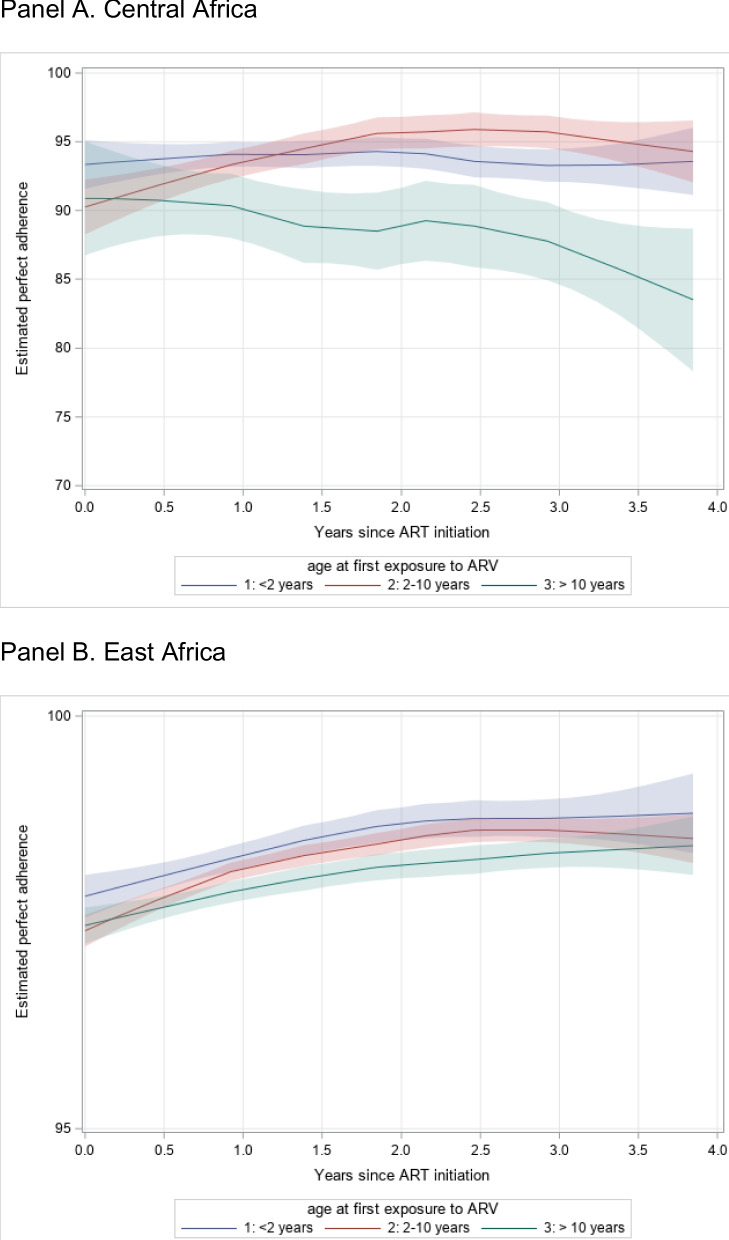
Adherence over time* in Central Africa and East Africa stratified by age at ART initiation. * Loess smoother and 95% confidence interval

Adherence was high according to self-reported adherence data (by clinician, patient, or caregiver), or pill counts, which were available for Central Africa and East Africa (all other sites used viral load measurement as their adherence measure). In Central Africa, patients’ adherence was assigned by clinician report as “bad,” “mediocre,” or “good,” of which 94% of encounters were classified as good adherence. Among sites in East Africa, clinics used 7 different adherence measures including self-reported missed doses or days with missed doses in the past 7 days and 30 days as well as qualitative classification. For comparison to Central Africa, among patients who were categorized as having “poor,” “fair,” and “good” adherence, 99% of encounters in East Africa sites were classified as good adherence. The most common adherence data at East Africa sites came from questions to caregivers about having any missed doses of ART in the past month, with 99% of encounters recording no missed doses in the past month. Over time, adherence levels by these measures tended to remain high over across age groups (< 2 years, 2–10 years, and > 10 years) in Central Africa and East Africa, although there was more variation in data from Central Africa especially among older children > 10 years (Fig. 2).

### Patterns and predictors of viral suppression over time

**Fig. 3:**
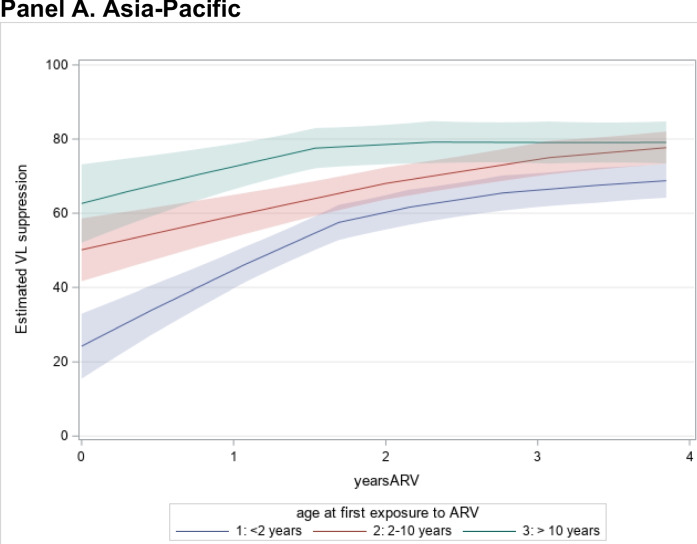
Virologic suppression by time on ART within IeDEA clinical sites in the Asia–Pacific, CCASAnet, East Africa, and Southern Africa

Viral suppression data were available from sites in four regions: Asia–Pacific, CCASAnet, East Africa, and Southern Africa (Fig. 3). Among patients at sites in the Asia–Pacific and Southern Africa, viral suppression rates increased rapidly after ART initiation and then plateaued afterward, with viral suppression levels remaining between 60–80% at two years after ART start. Viral suppression among children in CCASAnet region was between 20–50% at two years after ART initiation and stayed relatively constant over time. In East Africa, virologic suppression rates were between 40–50% at ART initiation and decreased gradually the longer the time since ART start, before starting to gradually increase.

In East Africa and Southern Africa, higher baseline CD4 counts were associated with a greater likelihood of viral suppression; however, in CCASAnet and the Asia–Pacific, we did not find a consistent relationship between CD4 count and viral suppression (Table 4). We also found evidence that very young children (< 2 years of age), older children (> 10 years of age), and males were less likely to experience viral suppression in East Africa and Southern Africa. In East Africa, children aged 2–10 years had about 65% higher odds of viral suppression compared to children > 10 years (aOR = 1.6, 95%CI 1.3,2.1), while males had 25% lower odds of viral suppression compared to females (aOR = 0.8 95%CI 0.6,0.95). In Southern Africa, children aged 2–10 years also had higher odds of viral suppression compared to children > 10 years (aOR = 1.3, 95%CI 1.1,1.5), and children aged < 2 years of age had lower odds of viral suppression compared to children > 10 years (aOR = 0.5, 95%CI 0.4,0.6). Males were also less likely to be virally suppressed (aOR = 0.9, 95%CI 0.8,1.0). In Asia–Pacific sites, children < 2 years of age had lower odds of viral suppression compared to children > 10 years (aOR = 0.3, 95%CI 0.2,0.5) but there was no relationship between sex and viral suppression. In the Asia–Pacific, CCASAnet, and Southern Africa, the likelihood of viral suppression increased by the number of years since ART start (40% annually in the Asia–Pacific and 35% in CCASAnet).

**Table 4:** Factors Associated with Viral Suppression

Factor	Asia Pacific *N* = 2,338	CCASAnet *N* = 686	East Africa *N* = 1,647	Southern Africa *N* = 10,030
	Adjusted Odds Ratios (95% CI)			
CD4 count				
50–99	1.0 (0.7,1.5)	1.6 (0.4,7.0)	1.6 (1.0,2.6)	1.0 (0.8,1.3)
100–199	0.8 (0.6,1.2)	1.1 (0.3,4.1)	1.3 (0.9,2.0)	1.1 (0.9,1.3)
200–349	1.2 (0.8,1.7)	2.0 (0.7,5.9)	1.9 (1.3,2.9)*	1.1 (1.0,1.3)
350–500	1.2 (0.8,1.8)	1.4 (0.5,4.2)	1.3 (0.8,2.1)	1.2 (1.1,1.5)*
> 500	0.7 (0.5,1.0)	0.7 (0.3,1.9)	2.9 (2.0,4.2)*	1.3 (1.1,1.5)*
< 50 (ref)	-	-	-	-
Age				
< 2 years	0.3 (0.2,0.5)*	0.7 (0.3,1.7)	1.0 (0.6,1.6)	0.5 (0.4,0.6)*
2–10 years	0.8 (0.6,1.1)	1.2 (0.5,2.6)	1.6 (1.3,2.1)*	1.3 (1.1,1.5)*
> 10 years (ref)	-	-	-	
Sex				
Male	0.9 (0.8,1.2)	1.0 (0.6,1.6)	0.8 (0.6,0.9)*	0.9 (0.8,1.0)*
Female (ref)	-	-	-	
Years since ART start	1.40 (1.37,1.42)*	1.35 (1.33,1.38)*	1.01 (0.97,1.07)	1.32 (1.28,1.36)*

Adjusted for age at ART, sex, and weeks since ART start

^*^Significant at *p* < 0.05

### Impact of viral suppression on loss to follow up and mortality

Across 4 IeDEA regions, viral suppression was associated with between 25%−48% lower likelihood of loss to follow up (aHR 0.55—0.75, *p*-value < 0.0001—0.0297). Children between 2–10 years, compared to children 10 years and older, had consistently lower likelihood of dropout (Table 5). Viral suppression was associated with a 4- to 15-fold lower likelihood of mortality (aHR 0.07—0.22, *p*-value < 0.0001 in all cases).

**Table 5:** Impact of viral suppression on loss to follow up and mortality

Factor	Asia–Pacific *N* = 2,259	CCASAnet *N* = 677	East Africa *N* = 1,619	Southern Africa *N* = 9,465
*Loss to follow up*	Adjusted Hazard Ratios (95% CI)			
Viral suppression (< 400 copies/mL)	0.5 (0.4,0.7)*	0.7 (0.6,1.0)*	0.5 (0.4,0.8)*	0.7 (0.6,0.8)*
CD4 count				
50–99	0.5 (0.3,0.9)*	1.1 (0.5,2.2)	0.6 (0.3,1.1)	1.1 (0.8,1.5)
100–199	0.5 (0.3,0.9)*	0.8 (0.4,1.6)	0.8 (0.5,1.3)	1.1 (0.8,1.4)
200–349	0.9 (0.6,1.3)	1.0 (0.6,1.7)	0.6 (0.4,1.0)*	1.0 (0.8,1.3)
350–500	0.6 (0.3,1.0)*	1.3 (0.7,2.1)	0.5 (0.3,0.9)*	1.0 (0.8,1.3)
> 500	0.7 (0.5,1.1)	0.3 (0.8,2.1)	0.5 (0.3,0.9)*	0.9 (0.8,1.2)
< 50 (ref)	-	-	-	-
Age				
< 2 years	1.0 (0.6,1.6)	0.3 (0.2,0.5)*	1.0 (0.5,2.0)	0.9 (0.7,1.1)
2–10 years	0.6 (0.4,0.9)*	0.5 (0.4,0.7)*	0.7 (0.5,0.9)*	0.9 (0.7,1.0)
> 10 years (ref)	-	-	-	
Sex				
Male	0.8 (0.6,1.1)	1.1 (0.9,1.5)	0.9 (0.7,1.3)	1.0 (0.8,1.1)
Female (ref)	-	-	-	
*Mortality*	Adjusted Hazard Ratios (95% CI)			
Viral suppression (< 400 copies/mL)	0.2 (0.1,0.3)*	0.1 (0.1,0.3)*	0.1 (0.0,0.2)*	0.2 (0.2,0.3)*
CD4 count				
50–99	0.5 (0.2,1.1)	0.5 (0.2,1.9)	1.0 (0.5,1.9)	0.7 (0.4,1.3)
100–199	0.6 (0.3,1.3)	0.4 (0.1,1.2)	0.5 (0.2,0.9)*	0.5 (0.3,0.8)*
200–349	0.5 (0.2,1.0)	0.4 (0.1,0.9)*	0.5 (0.3,1.0)*	0.4 (0.3,0.7)*
350–500	0.6 (0.3,1.4)	0.3 (0.1,0.9)*	0.3 (0.1,0.6)*	0.6 (0.4,1.0)
> 500	0.2 (0.1,0.5)*	0.1 (0.1,0.3)*	0.2 (0.1,0.4)*	0.3 (0.2,0.5)*
< 50 (ref)	-	-	-	-
Age				
< 2 years	0.8 (0.4,1.9)	0.7 (0.3,1.7)	5.0 (2.4,10.8)*	1.5 (0.9,2.3)
2–10 years	0.5 (0.3,0.8)*	0.5 (0.2,1.0)	1.4 (0.9,2.3)	0.5 (0.3,0.8)*
> 10 years (ref)	-	-	-	
Sex				
Male	1.2 (0.7,1.9)	0.8 (0.5,1.5)	1.4 (0.9,2.2)	1.1 (0.8,1.5)
Female (ref)	-	-	-	

^*^Significant at *p* < 0.05

## Discussion

This global analysis reveals critical needs for long-term pediatric and adolescent HIV care. As adolescents continue to have increasing rates of mortality due to HIV, finding strategies to maintain and support global adherence to ART and continued clinical follow-up must not be forgotten, especially for those growing up with HIV across their developmental lifespan.

Our findings from the global survey assessing pediatric HIV care sites shows routine evaluation of ART adherence through clinician assessments or pharmacy refills, but sites seldom use validated measures and may rely on viral load measurements as a proxy for adherence measurement. This means that many programs struggle to separate challenges with ART adherence and clinic engagement from treatment failure due to evolving viral resistance. In East Africa, the one region for which both validated adherence measures and viral load measurements could be generally compared (Figs. 2 vs 3), differences in the rates of reported adherence and viral suppression can be seen. Specific studies to validate adherence measures and examine reported adherence, viral suppression and viral resistance in East Africa have been conducted recently and preliminary results point to the insufficiency of using viral load measurements as a proxy for adherence [11, 12]. While these global survey results were detailed in 2015, reductions in the funding for counseling and adherence support services available through global funders have substantially decreased since that time [13–15], so there is no reason to think that sites have substantially increased the services provided. Scaling up the use of validated self-report measures for children’s ART adherence would offer more accurate data related to challenges children and families may face with adherence.

The four regions with viral load data for patients had variable viral suppression over time, the association between CD4 count and viral suppression, and which populations have greater odds of viral suppression. However, viral suppression was consistent with good adherence overall. As previously described, Asia–Pacific, CCASAnet, and Southern Africa sites only used viral load to track adherence, but clinicians must remember it is possible to have viral failure with good adherence due to drug resistance. While viral suppression is a critical outcome, using it as the exclusive measure of treatment success may not allow clinicians to determine whether treatment failures result from non-adherence or HIV drug resistance. Moreover, replacing CD4 testing with viral load testing means that current immune status may not be fully known. There also remains significant variation globally in whether viral load testing is routinely available [16]. The regional differences in achieving viral suppression, where Southern Africa has more rapid achievement of viral suppression and at higher rates of suppression also bears consideration. As reflected in the survey data, the Southern Africa sites generally had more resources for pediatric care and for adherence support available than the other African regions, as well as a longer track record of providing ART and more rapid access to newer ART regimens. These important regional differences in the care systems may be reflected in the trends towards more rapid viral suppression.

Some of the data, specifically in East and Southern Africa, suggest that older children (> 10 years old) were less likely to experience viral suppression compared to younger children. These young adolescents were also more likely to drop out of care and experienced higher mortality. These findings highlight the substantial risks currently faced by this currently “aging” population of adolescents, as they are likely to continue to have poorer retention in care, lower rates of virologic suppression, and higher rates of mortality compared to their pediatric and adult counterparts [17–23]. The global needs of adolescents living with HIV, particularly those infected through vertical transmission and more likely to have advanced HIV disease, continue to require urgent attention. In addition, two other subsets of children, those under 2 years and males, were also less likely to have viral suppression. These findings may point to specific risks in these groups that should also be considered. The challenges with adequate ART formulations to support adherence to infants may be an ongoing issue in achieving viral suppression, even as we see smaller numbers of infants living with HIV where prevention of perinatal transmission is accessible. The differences in viral suppression by gender and how strategies to support adherence might be better tailored for male children and youth merits further consideration.

These data provide detailed understanding of adherence globally, but there are a number of limitations as well. As described, the sites used heterogeneous methods of adherence measurement with variable validity. This lack of uniformity in data collection prohibits a clear comparison between regions. Data were collected from 2002 to 2015; and therefore, may be outdated and not representative of current adherence data. However, patterns of medication-taking and supports for adherence remain significant for understanding both resistance patterns and health behaviors, no matter what regimen a child is on. In addition, the extent of non-adherence seen here may have implications for further regimens, even in the era of integrase inhibitors. Studies suggest that prior NNRTI resistance is associated with long-term failure of integrase inhibitor-containing first-line regimens [24]. While many sites reported access to drug resistance testing, probing revealed this was typically accessible only through research studies or special government permissions, matching current global assessments highlighting gaps in resistance data for children with HIV [25]. Without drug resistance testing, we cannot identify differences in non-adherence, viral failure, and drug resistance data among the regions. Such data would allow us to better understand treatment failure and non-adherence, no matter what regimen. Despite limitations, the large scope of this cohort, with six global regions and tens of thousands of pediatric patients – one of the world’s largest cohorts of children and youth living with HIV after vertical transmission, remains significant for characterizing the global pediatric populations living with HIV. Moreover, adherence to ART only increases in significance as time goes on, with the evolution of viral resistance ongoing, a critical challenge for global populations who may not have access to additional ART regimens.

Using historical cohort data to understand the scope of global ART non-adherence helps forecast the subsequent global impact on regimen selection and the need for expanded regimen options [26]. Critical research gaps related to adherence and drug resistance among children and adolescents remain. As we move closer to a time when long-acting and/or injectable ART options become available for adolescents and younger children, we will still need strong engagement with the health system distributing the medications and to find effective strategies to incorporate the needs of children and their families into treatment decisions and case management. Adherence to medications and engagement in clinical care remain crucial to prevent HIV viral resistance, transmission, and mortality among children living with HIV – and to stemming the HIV pandemic.

## Conclusions

Finding effective adherence interventions remains a global priority and regional priorities can be guided by this comprehensive global picture of adherence-related measurement and support.

## Supplementary Information

Supplementary Material 1.

## Data Availability

The data that support the findings of this study are available on request from the corresponding author. All data requests must be approved by IeDEA.

## Abbreviations

ART: Antiretroviral Therapy
IeDEA: International Epidemiology Databases to Evaluate AIDS
HIV: Human Immunodeficiency Virus
CCASAnet: Caribbean, Central and South America
