# Supplementary material for: Rates of adherence, adherence measurement, and support services for children and adolescents living with HIV followed in global sites of the International Epidemiology Databases to Evaluate AIDS (IeDEA)
Source: BMC Pediatr. 2025 Oct 2;25:706. doi: 10.1186/s12887-025-05939-4 (PMC12492743; doi:10.1186/s12887-025-05939-4)
Supplement: Supplementary file 1 — Supplementary Material 1. [file 12887_2025_5939_MOESM1_ESM.docx]

**Supplemental Table. Adherence and Virologic Outcomes**

| **Adherence Measure** | **Asia-Pacific** | **CCASAnet** | **Central Africa** | **East Africa** | **Southern Africa** | **West Africa** |
| --- | --- | --- | --- | --- | --- | --- |
| Number of patients with at least one adherence measure | 3,114 (71%) | 957 (55%) | 905 (99%) | 11,898 (100%) | 11,960 (38%) | 0 (0%) |
| Median number of adherence measures per patient | 6 | 15 | 21 | 25 | 4 | - |
| Median time (days) between adherence measurements per patient | 175 | 126 | 33 | 35 | 173 | - |
| Adherence measures | Viral load | Viral load | Clinician-reported | Multiple measures | Viral load | - |
